# A review of the Rett Syndrome Behaviour Questionnaire and its utilization in the assessment of symptoms associated with Rett syndrome

**DOI:** 10.3389/fped.2023.1229553

**Published:** 2023-07-28

**Authors:** Alan K. Percy, Jeffrey L. Neul, Timothy A. Benke, Eric D. Marsh, Daniel G. Glaze

**Affiliations:** ^1^ Department of Pediatrics, University of Alabama at Birmingham, Birmingham, AL, United States; ^2^ Vanderbilt Kennedy Center, Vanderbilt University Medical Center, Nashville, TN, United States; ^3^ Children’s Hospital of Colorado/University of Colorado School of Medicine, Aurora, CO, United States; ^4^ Department of Neurology, Children’s Hospital of Philadelphia, Philadelphia, PA, United States; ^5^ Texas Children’s Hospital/Baylor College of Medicine, Houston, TX, United States

**Keywords:** Rett Syndrome Behaviour Questionnaire, trofinetide, caregiver, healthcare providers, neurobehavioral symptoms

## Abstract

The Rett Syndrome Behaviour Questionnaire (RSBQ), which is completed by the caregiver, is one of the most widely used efficacy measures in clinical studies of Rett syndrome (RTT) due to its specificity to the core features of RTT. As healthcare providers participate in routine healthcare assessments of individuals with RTT in clinical practice, there is a need for these providers to understand the psychometric properties of the RSBQ and how it relates to the core clinical features of RTT. Here, we describe the characteristics of the RSBQ, review the literature on its validity and reliability as well as its performance in a phase 2 study and the recent phase 3 LAVENDER study. The RSBQ was first shown to discriminate RTT from other intellectual disorders with good inter-rater and test–retest reliability scores. It was subsequently validated as an appropriate instrument for measuring behavior in females with RTT and adopted as a clinical trial outcome. In LAVENDER, the FDA-approved drug trofinetide significantly improved the RSBQ total score over placebo in girls and women with RTT and change from baseline for all RSBQ subscores were directionally in favor of trofinetide. The change in RSBQ was aligned with the Clinical Global Impression-Improvement scale, suggesting that improvement in behavioral components may be related to overall clinical status. Given its validity and ubiquity in RTT clinical studies, it is important that the interplay of the domains and the psychometric profile of the RSBQ are understood.

## Introduction

Rett syndrome (RTT) is a profoundly disabling neurodevelopmental disorder predominantly affecting females (1–3) and is primarily caused by mutations in the gene encoding X-linked methyl-CpG-binding protein 2 (MeCP2) (4).

Individuals with RTT undergo a period of apparently normal development during the first 6 months of life followed by developmental regression between 12 and 30 months of age characterized by partial or complete loss of spoken language and hand function skills, impaired or absent gait, and the development of repetitive hand stereotypies, which all represent the fundamental diagnostic criteria for RTT (5). Other common features include epilepsy, breathing disruptions while awake, gastrointestinal difficulties, autonomic abnormalities, scoliosis, and limited nonverbal communication skills (e.g., intense eye communication) (5–8). Behavior disturbances occur in nearly all individuals with RTT across their lifespan, with internalizing behaviors such as anxiety or mood swings being much more common overall than externalizing behaviors such as aggression, hyperactivity or self-injury, with anxiety-like behavior considered a significant parental concern (9, 10). Recommendations for regular annual assessments in the primary care setting include those related to behavior such as anxiety and depression (11).

A number of caregiver- and clinician-assessed measures have been developed to assess disease severity, functional ability, and primarily neurological and sociobehavioral abnormalities in clinical studies. The Rett Syndrome Behaviour Questionnaire (RSBQ) assesses the severity of neurobehavioral problems from the perspective of the caregiver and is one of the most widely used measures due to the specificity of its psychometric profile to the core features of RTT and its acceptance by the United States Food and Drug Administration (FDA) for use in RTT studies.

In this review, we will provide a summary of the role for caregivers and healthcare providers in the management of RTT as well as describe the characteristics of the RSBQ and the association between its domains and clinical features of RTT, its reliability (sensitivity and specificity), and performance in a phase 2 study (12) and the recent phase 3, placebo-controlled LAVENDER study (Clinicaltrials.gov: NCT04181723) (13, 14) in girls and women with RTT aged 5–20 years.

## The role of the caregiver and healthcare provider

Primary care providers and other healthcare professionals caring for individuals with RTT are required to manage the evolving medical comorbidities of RTT effectively throughout an individual's lifespan, which can last beyond 50 years of age (15–17); however, many have limited first-hand experience in managing the disorder due to its rare occurrence (11).

Recent consensus guidelines for primary care providers recommend regular medical assessments (follow-up visits at least every 6 months) to screen for issues that can appear quickly, progress rapidly, and require intervention (11). In terms of behavioral assessments, it is recommended that primary care providers regularly screen for symptoms of anxiety and depression, such as withdrawal, screaming, and irritability. Respiratory assessments include screening for awake-disordered breathing (hyperventilating, breath holding, color change) and air swallowing (Table 1). Neurological assessments should include screening for abnormal movements (stereotypies and dystonia) and their level of impact on daily activities, fine motor skills (hand use), and gross motor skills (sitting, standing, and walking) (Table 1).

**Table 1:** Summary of consensus guidelines for primary care providers limited to the management of neurobehavioral symptoms in RTT (11).

Area of assessment	Assessment details	Frequency of assessment
Respiratory	• Screen for awake-disordered breathing (hyperventilating, breath holding, color change) and air swallowing	Annual wellness visit
Neurology	• Encourage follow-up with neurologist routinely; every 6 months if treated for seizures • Screen for abnormal movements (stereotypies and dystonia) and level of impact on daily activities	Annual wellness visit Primary care every 6 months is recommended to screen for issues that can appear quickly, progress rapidly, and require intervention
Developmental milestones	• Developmental regression (reduced hand use and language) typically stops between 2 and 3 years • Documentation of baseline, gains and losses of milestones • Fine motor: hand use (raking grasp, pincer grasp, rake, holding cup or spoon) • Gross motor: sitting, standing, and walking • Language: coo, babble, laugh, words • Engagement of regular and appropriate educational and specific therapies (physical, occupational, and speech)	Annual wellness visit
Communication	• Screen communication methods used by family and school: eye pointing, vocalizations, switches, iPad, eye gaze device	Annual wellness visit
Psychological/behavioral	• Screen for symptoms of anxiety and depression, such as withdrawal, screaming, and irritability • These may become more prominent with age or in individuals with milder clinical presentations • Behavioral inconsistency is typical and may be affected by physical factors such as sleep or environment; assess for intolerance of excessive stimuli (i.e., bright lights, loud noises) • Enquire about sensory processing difficulties	Annual wellness visit Primary care every 6 months is recommended to screen for issues that can appear quickly, progress rapidly, and require intervention
Sleep	• Review sleep initiation, staying asleep, snoring or coughing, and frequency of nocturnal interventions by caregivers • Review safety of bed and bedroom • Consider laboratory evaluation for iron deficiency if concerns arise about disrupted sleep or restless leg syndrome: ferritin, serum iron, total iron binding capacity, transferrin	Annual wellness visit Primary care every 6 months is recommended to screen for issues that can appear quickly, progress rapidly, and require intervention

Adapted from Fu et al., 2020, which provides the full list of consensus guidelines (11).

Consensus guidelines on managing Rett syndrome across the lifespan by Fu C, Armstrong D, Marsh E, et al. is licensed under CC BY-NC 4.0, published by BMJ 2020.

Caregivers are tasked with managing daily activities and communicating with the individual with RTT on a daily basis. Together with neurologists and pediatric neurologists, caregivers are primarily responsible for relaying feedback on clinical assessments in trials and determining improvement in the individual with RTT; this emphasizes the need to address the top concerns of caregivers, who often cite communication as being one of the most important (18–20).

Understanding the natural history of these core symptoms in RTT is critical to directing diagnosis, guiding prognosis, and testing the efficacy of new interventions in clinical trials. As an established efficacy measure in RTT clinical trials, it is important that healthcare providers and caregivers are able to interpret the psychometrics of the RSBQ.

## The RSBQ

The RSBQ is a validated assessment tool that has been accepted by the FDA as a primary outcome measure for clinical studies in RTT and has been used on numerous occasions to assess symptoms in clinical trials in RTT (12, 13, 21, 22). The RSBQ is a caregiver-completed scale assessing a wide range of neurological and behavioral symptoms in RTT that has been used across a range of ages (2–47 years) in clinical studies (23–25). The RSBQ consists of 45 items of which 38 items are grouped into 8 domains/subscales that reflect the core features of RTT: General Mood; Breathing Problems; Hand Behaviors; Repetitive Face Movements; Body Rocking and Expressionless Face; Nighttime Behaviors; Fear/Anxiety; and Walking/Standing) (Table 2). When the RSBQ was first developed, each item was grouped into the appropriate subscale based on a factor analysis (26); the 7 items that did not belong under any of the subscales were classed as “uncategorized” but contribute to the overall total score. Each item is rated on a Likert scale as 0 (behavior “not true”), 1 (behavior “somewhat or sometimes true”) or 2 (behavior “often true”), with the total score ranging from 0 to 90 (higher scores indicate increased severity) (26).

**Table 2:** The Rett Syndrome Behaviour Questionnaire (26).

Domain/subscale			
General Mood (8 items; max score = 16)	Breathing problems (5 items; max score = 10)	Hand behaviors (6 items; max score = 12)	Repetitive face movements (4 items; max score = 8)
• Spells of screaming for no apparent reason during the day • Abrupt changes in mood • Certain days/periods where she performs much worse than others • There are times when she appears miserable for no apparent reason • Screams hysterically for long periods of time and cannot be consoled • There are times when she is irritable for no apparent reason • Spells of inconsolable crying for no apparent reason during the day • Vocalizes for no apparent reason	• There are times when breathing is deep and fast (hyperventilation) • There are times when breath is held • Air or saliva is expelled from mouth with force • Swallows air • Abdomen fills with air and sometimes feels hard	• Does not use hands for purposeful grasping • Hand movements are uniform and monotonous • Has frequent naps during the day • Restricted repertoire of hand movement • Has difficulty in breaking/stopping hand stereotypies • The amount of time spent looking at objects is longer than the time spent holding or manipulating them	• Makes repetitive movements involving fingers around tongue • Makes mouth grimaces • Makes repetitive tongue movements • Makes grimacing expressions with face
Domain/subscale			
Body rocking and expressionless face (6 items; max score = 12)	Nighttime Behaviors (3 items; max score = 6)	Fear/Anxiety (4 items; max score = 8)	Walking/Standing (2 items; max score = 4)
• Expressionless face • Seems to look through people into the distance • Uses eye gaze to convey feelings, needs, and wishes • Rocks self when hands are prevented from moving • Tendency to bring hands together in front of chin or chest • Rocks body repeatedly	• Spells of screaming for no apparent reason during the night • Spells of laughter for no apparent reason during the night • Spells of inconsolable crying for no apparent reason during the night	• Spells of apparent anxiety/fear in unfamiliar situations • Seems frightened when there are sudden changes in own body position • There are times when parts of body are held rigid • Spells of apparent panic	• Although can stand independently tends to lean on objects or people • Walks with stiff legs
Uncategorized (not included in subscales but part of the total score)			
• Spells of laughter for no apparent reason during the day • Has wounds on hands as result of repetitive hand movements	• Shifts gaze with slow horizontal turn of head • Makes repetitive hand movements apart	• Appears isolated • Grinds teeth	• Vacant “staring” spells

Adapted from The Rett Syndrome Behaviour Questionnaire (RSBQ): refining the behavioural phenotype of Rett syndrome. Mount et al. (26). Copyright © 2002 Association for Child and Adolescent Mental Health. Reproduced with permission of John Wiley & Sons Inc.

The percentage occurrence of potentially characteristic behaviors in RTT include hand stereotypies, which are almost universal (99%), teeth grinding (58%), sleeping difficulties and nighttime laughing (64%), anxiety or inappropriate fear (73%), low mood/changeable mood (77%), breath holding (63%), and hyperventilation (77%) (25), which reflect many of the caregiver concerns and are all captured in the domains of the RSBQ.

The heterogeneity of the RTT phenotype presents a challenge both in clinical trial assessments and routine clinical practice as often only a limited set of difficulties may predominate at any given time; however, the total scale scores for the RSBQ are influenced by multiple items, some of which are neurologic and others behavioral in nature, and it is this broad scope that allows changes in an individual feature to be captured whether it be related to physical functioning or behavior.

The RSBQ was initially developed as a diagnostic tool to differentiate females with RTT from those with severe intellectual disability after it was shown to be highly discriminatory between individuals with RTT (*n* = 143) and intellectual disability (*n* = 85) (26). The RSBQ transitioned to a clinical trial outcome assessment after it was shown to adequately describe behavioral characteristics in RTT in a UK and Australian cohort (23). In a subsequent study in 74 girls with RTT, the Fear/Anxiety subscale was deemed reliable and valid for use in clinical and research settings (24). The RSBQ was first used in a placebo-controlled clinical trial to investigate the efficacy of mecasermin (recombinant human insulin-like growth factor 1) as a treatment for RTT though, together with most of the study outcomes, failed to show a statistically significant difference between drug and placebo (22).

The RSBQ adds clinical context by indicating severity and frequency of symptoms, is relatively simple to complete, and is correlated with functioning (23–25). More research is needed to improve understanding of the correlation of RSBQ domains with the overall score, the floor or ceiling effects due to the limited range between the absence and presence of a characteristic, and a minimal clinically important difference. While the RSBQ addresses most of the features of RTT, there are important clinical domains that are common in RTT including gastrointestinal and nutritional problems (27, 28), and scoliosis (29), that are not covered by the RSBQ.

The number of items and range of scores in a subscale is important in terms of the validity and reliability of a psychometric profile. The domains/subscales of the RSBQ and their clinical relevance in the diagnosis and assessment of disease severity are summarized below.

### General mood: 8 items (maximum score = 16)

The General Mood domain includes items related to unexpected periods of crying, screaming, irritability, and vocalization that are characteristic in RTT particularly during the regression period in early childhood (<5 years) and is the most weighted (due to the number of items) of all 8 domains. Abrupt changes in mood associated with spells of screaming, crying, and being irritable or miserable for no apparent reason were shown to be speciﬁc to RTT (26). In 3 surveys in RTT, low mood or mood changes were reported in 66%–77% of individuals (25, 30, 31).

### Breathing problems: 5 items (maximum score = 10)

Breathing problems are RTT-specific and are one of the supportive diagnostic criteria (5); items in this domain of the RSBQ are related to hyperventilation, air swallowing, and breath holding (26). The autonomic dysregulation in RTT manifests most prominently as an irregular breathing pattern that affects almost all individuals with RTT during their lifetime (8). In most individuals breathing problems present by approximately age 4 and are characterized by rapid breathing (hyperventilation), breath holding, and/or air swallowing that fluctuate in intensity while awake and disappear during sleep (8, 32). This domain is essentially a neurological and not a behavioral assessment and is shown to correlate with clinical severity as measured using the RTT Severity Scale, which assesses overall clinical severity and 7 individual parameters: frequency and manageability of seizures; respiratory irregularities; scoliosis; ability to walk; hand use; speech; and sleep (33).

### Hand behaviors: 6 items (maximum score = 12)

This domain is a RTT-specific, neurology-related component of the RSBQ that includes hand stereotypies (involuntary, coordinated, repetitive movements) and loss of purposeful hand function, which are hallmark features of RTT and part of the main diagnostic criteria of typical RTT (5). Based on data from the RTT Natural History Study, hand stereotypies affect every individual with typical RTT and >96% with atypical RTT (34).

### Repetitive face movements: 4 items (maximum score = 8)

This domain could be considered part of the spectrum of stereotypical movements in RTT, or a manifestation of extrapyramidal features (23) and assesses repetitive mouth and tongue movements and facial grimacing (26). Repetitive face movements are reported to be more frequent or severe in individuals with RTT compared with those with other severe intellectual disability (26), which may aid in the differential diagnosis of RTT.

### Body rocking and expressionless face: 6 items (maximum score = 12)

This domain is heterogeneous in its inclusion of both eye gaze and expressionless face with the former included in the supportive criteria for atypical RTT (5). Eye gaze and use of “eye pointing” to communicate are features that can help distinguish RTT from other causes of severe intellectual disability (23) and thus is an important diagnostic feature. Rocking movements and expressionless face, which are less defined and overlap with features of autism, have been described in 16% and 29% of individuals with RTT, respectively (35). In the recent phase 3 LAVENDER study investigating the efficacy and safety of trofinetide treatment in RTT, the scoring for one of the items from this domain (“uses eye gaze to convey feelings, needs, and wishes”) was reversed (i.e., 2 minus the observed item score). This was to reflect the fact that higher scores for this item indicate a positive benefit in the ability to communicate (14).

### Nighttime behaviors: 3 items (maximum score = 6)

Each of the 3 items in this domain relate to episodes of crying, laughing, or screaming during the night, which are recognized features of RTT (5). Sleeping difficulties and nighttime laughing have been reported in 21%–84% of individuals in 5 surveys of RTT (25, 30, 31, 35, 36). This behavior is more frequent or severe in individuals with RTT compared to those with other severe intellectual disability (26) and is thus another useful domain for the differential diagnosis of RTT. In a recent survey of 287 caregivers of individuals with RTT who were asked to complete the sleeping questionnaire for children with neurological and other complex diseases (SNAKE), sleep quality was rated as very good to good by over 60% of caregivers, which contrasts with the data available in the literature (37). Behavioral disorders were also assessed using the RSBQ, and those related to regression, such as loss of acquired hand skills (*p* = 0.046) and isolation (*p* = 0.002), were found to be significantly associated with sleep quality.

### Fear/Anxiety: 4 items (maximum score = 8)

This feature of RTT could be related to autonomic dysfunction and while it is not recognized among the criteria used to diagnose RTT, manifestations of fear and anxiety are commonly observed in individuals with RTT. In 4 surveys of RTT, anxiety or inappropriate fear was reported in 68%–75% of individuals (25, 30, 31, 36). This domain is also important in differential diagnosis since it is more frequent or severe in individuals with RTT compared to those with other severe intellectual disability (26). In a study that examined the profiles of anxious behavior in 74 girls with RTT (24), the severity of general anxiety was inversely correlated with clinical severity assessed using the RTT-Clinical Severity Scale, which includes 13 items specific to the RTT phenotype that measure historical and current clinical severity (38). Current consensus guidelines recommend routine screening for symptoms of anxiety and depression, such as withdrawal, screaming, and irritability (11).

### Walking/Standing: 2 items (maximum score = 4)

The Walking/Standing domain of the RSBQ captures gait abnormalities, 1 of the 4 key criteria of RTT (5), and characterizes the manifestation of the motor impairment and spasticity associated with RTT (23). The Walking/Standing domain has been observed to have the lowest level of positive intercorrelation with the other domains (25), which could be attributed to the fact that the level of functional ability that is captured with this domain is often inversely correlated with behavioral problems (23). This inverse correlation also applies when considering the relationship of this domain with clinical severity. In a survey of 91 girls and women with RTT the Walking/Standing domain was the only domain that was significantly associated with severity based on the Simplified Severity Score, whereby scores were higher among those with less severe clinical characteristics (25). The Simplified Severity Score evaluates the overall severity of RTT and indicates domains (sitting, walking, hand use, speech, epilepsy, and spine deformation) considered to influence evolution and severity in the long term (39). This imbalance of functioning and behavioral aspects captured in the RSBQ emphasizes the importance of complementary outcome measures such as the Clinical Global Impression-Improvement (CGI-I) scale (38) in clinical trials. The CGI-I assesses how much the affected individual's illness (RTT as a whole) has improved or worsened relative to a baseline state on a 7-point scale (1 = very much improved, 2 = much improved, 3 = minimally improved, 4 = no change, 5 = minimally worse, 6 = much worse, 7 = very much worse) (38).

In summary, all of the RSBQ domains are clinically relevant to the core features of RTT. Despite evidence that increased clinical severity significantly predicts increased RSBQ total scores (33), significant positive correlations between individual domains and clinical severity have only been demonstrated for Breathing Problems, while inverse correlations are observed for Fear/Anxiety (24) and Walking/Standing (25). It is, however, worth appreciating that clinical severity was assessed in those studies using different scales (i.e., Simplified Severity Score, RTT-Clinical Severity Scale, and RTT Severity Scale), which themselves might lack sensitivity to the progression of RTT over time (i.e., Simplified Severity Score (25)) or have other have psychometric limitations.

## Validity and reliability of the RSBQ

Studies have investigated the reliability of the RSBQ (test–retest reliability, intra-rater reliability), validated its discriminatory diagnostic potential in a RTT population versus a population with intellectual disability, and compared behavior profiles in UK and Australian populations (23, 26). Moderate to high internal consistency was reported for the total score and the 8 subscales, with good inter-rater and test–retest reliability scores, and significantly higher scores in a RTT population versus those with intellectual disability, thus validating its use as a diagnostic tool (26). The Cohen's *d* effect sizes were all large (40), the smallest being 0.81 for the Walking/Standing domain and the largest for Hand Behaviors (2.24) suggesting that Hand Behaviors is the domain with the greatest specificity to RTT. Hand behaviors and breathing problems were virtually only present in individuals with RTT, while mood fluctuations, fear/anxiety behaviors, inconsolable crying and screaming at night, and repetitive mouth and tongue movements and grimacing were more frequent or severe in RTT compared to those with intellectual disability (26). Physical ability was significantly associated with the domains for Hand Behaviors and Walking/Standing (26); thus, these domains provide an additional assessment of physical functioning. Variability in each domain ranged from 4.6% for Walking/Standing and up to 11.9% for General Mood (26), which may be affected by the number of items in each domain. There was also good correlation in terms of the behavior profile identified using the RSBQ scores between Australian and UK populations with RTT (23). In addition, a recent examination of meaningful changes in RSBQ symptoms offered insights into the essential domains of life experiences of children with Rett syndrome and their caregivers (41).

Cianfaglione et al. (2015) analyzed potential differences in the RSBQ based on mutation and clinical severity in a UK national sample of 91 girls and women with RTT and showed that the Hand Behaviors domain contributed the most to the total score (25). Means for each domain were generally near half of the maximum scores possible and ranges were broad, suggesting an absence of scoring to the floor/ceiling and variability in the manifestation of the behavioral phenotype across individuals. There was a high degree of positive intercorrelation between all RSBQ domains other than the Walking/Standing domain. Barnes et al. investigated the reliability of the Fear/Anxiety domain in 74 girls with RTT and showed that internal consistency was comparable with that reported by Mount et al. (26), that age did not affect scores, and that severity of general anxiety was inversely correlated with clinical severity (RTT-Clinical Severity Scale) (24).

Despite evidence of a positive discriminatory benefit with the RSBQ in RTT, in a recent assessment of the RSBQ, it was reported that half of the items tested exhibited floor or ceiling effects (42). However, this study was itself criticized for inadequate representation of the population, the use of a clinically heterogeneous sample, and missing psychometric evaluations (43). In their rebuttal, the authors recommended that efforts should be directed to improve metrics for specific functions (walking, hand use, and nonverbal communication) and disabilities (seizures, sleep dysfunction, and gastrointestinal comorbidities) that caregivers routinely highlight as primary concerns in order to improve caregivers' interpretation of clinically meaningful improvements in RTT (44).

## Interpretation of RSBQ scores in clinical studies

The efficacy and safety of trofinetide, a synthetic analog of a tripeptide (glycine-proline-glutamate) derived from the N-terminus of insulin-like growth factor 1 (45) and the first drug to be approved for the treatment of RTT, was investigated in a placebo-controlled, phase 2 study (12), and the phase 3, placebo-controlled, LAVENDER study (13, 14).

**Figure 1:**
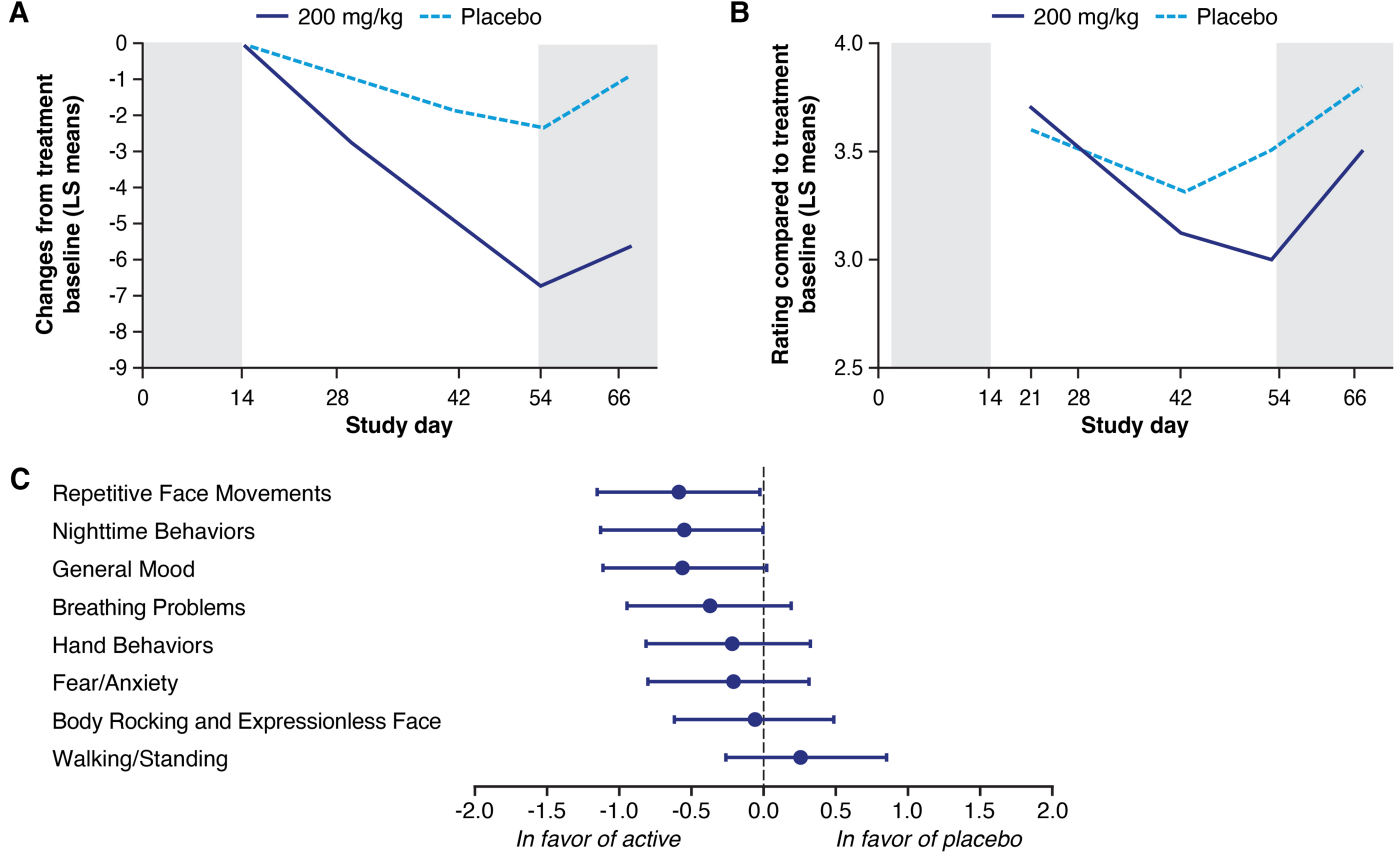
Least squares (LS) mean change in the Rett Syndrome Behaviour Questionnaire (RSBQ) (**A**) and Clinical Global Impression-Improvement (CGI-I) (**B**) following treatment with trofinetide (200 mg/kg) or placebo in the phase 2 study, and the change in the individual domain scores from the RSBQ (**C**). The RSBQ used for the phase 2 study was slightly modified as described by Kaufmann et al. (33) and included 39 items grouped into the 8 subscales; in panels **A** and **B**, shading at Day 0–14 represents the single-blind placebo, and shading beyond Day 54 the post-treatment follow-up. Glaze et al. (12). Copyright © 2019, American Academy of Neurology. Published by Wolters Kluwer.

In the phase 2 study, treatment with trofinetide at 200 mg/kg twice daily for 6 weeks showed statistically significant improvements relative to placebo on the RSBQ (Figure 1A) and was supported by improvements on overall functioning as measured by the CGI-I (Figure 1B). All of the domains except Walking/Standing were directionally in favor of the 200 mg/kg treatment group, with notable improvement in mood dysfunction and disruptive behavior (General Mood domain, *p* = 0.007), breathing problems (Breathing Problems domain, *p* = 0.095), and repetitive movements (Repetitive Face Movement domain, *p* = 0.047) (Figure 1C). The RSBQ total score had medium Cohen's *d* effect sizes (−0.487) that were similar to the CGI-I (−0.645) (12).

**Figure 2:**
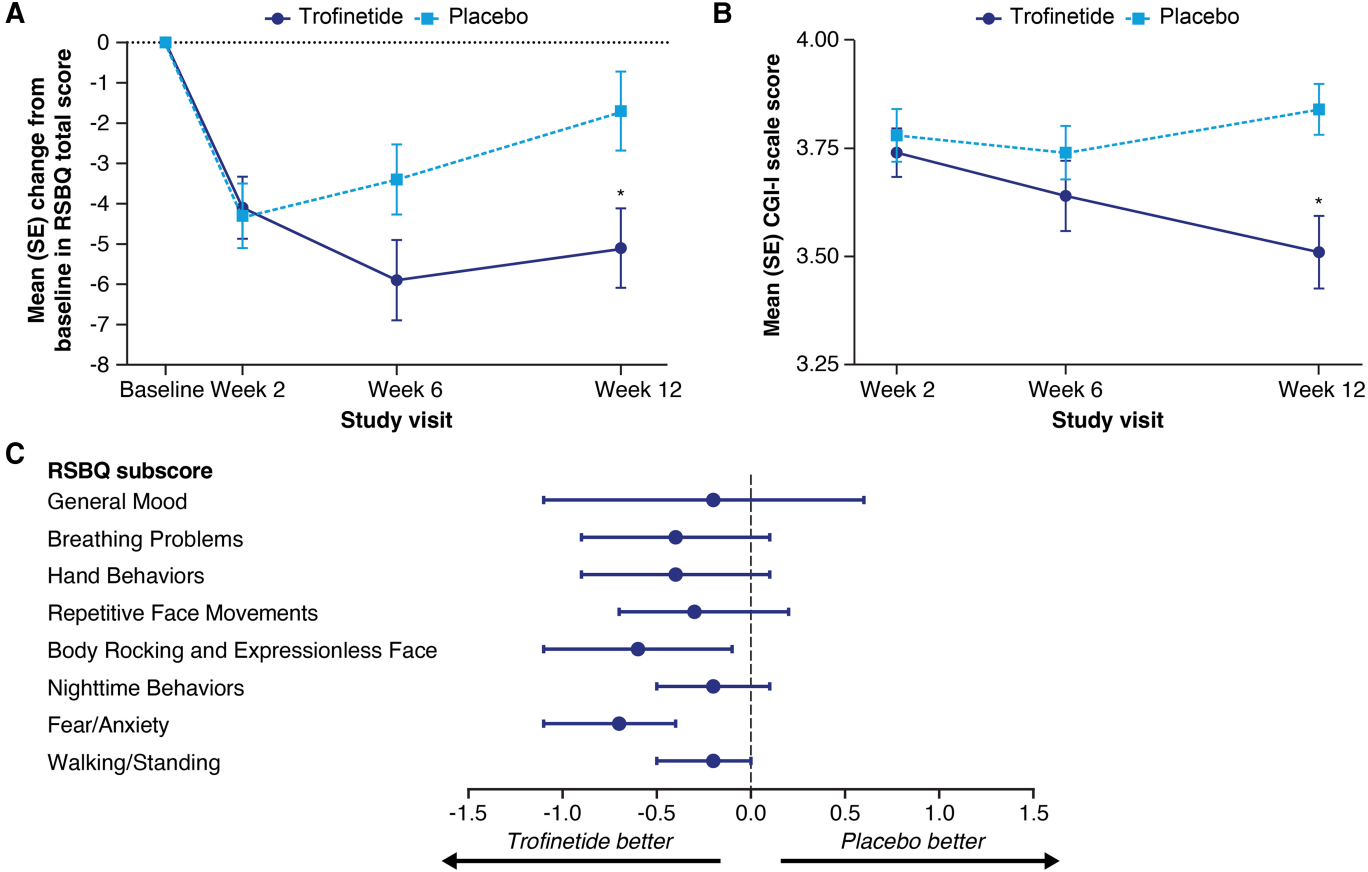
Mean (SE) change from baseline in the Rett Syndrome Behaviour Questionnaire (RSBQ) total score (**A**) and mean (SE) Clinical Global Impression-Improvement (CGI-I) scale score (**B**) at each study visit, and least squares mean treatment differences with 95% confidence interval (CI) for the change in RSBQ domain scores (**C**) in the phase 3 LAVENDER study. In panels **A** and **B**, asterisks denote significance (*p* ≤ 0.05) based on the least squares mean difference from the mixed-effect model for repeated measures analysis. In panel **C**, CI widths have not been adjusted for multiplicity. The score for item 31 (“uses eye gaze to convey feelings, needs, and wishes”) was reversed in the calculations of the RSBQ total score and subscores. Figures adapted from Neul et al. (14). Copyright (open license CC BY) Springer Nature 2023.

In the LAVENDER study, which included females with RTT aged 5–20 years, after 12 weeks' treatment, twice-daily trofinetide demonstrated statistically significant differences from placebo for both coprimary endpoints (RSBQ and CGI-I) (14). Mean (standard error, SE) change from baseline to week 12 in the RSBQ total score was −5.1 (0.99) and −1.7 (0.98) in the trofinetide and placebo group, respectively. The least squares (LS) mean treatment difference was −3.1 (95% CI, −5.7 to −0.6; *p* = 0.0175, Cohen's *d* effect size = 0.37; Figure 2A). The change in RSBQ was aligned with the CGI-I using RTT-specific anchors (38), suggesting that improvement in behavioral components is predictive of overall clinical status (Figure 2B). Cohen's *d* effect sizes for the coprimary endpoints fell in the 0.4–0.5 medium effect size range (0.37 for the RSBQ, 0.47 for the CGI-I) (40). The changes from baseline for all RSBQ domains were all directionally in favor of trofinetide (Figure 2C), with notable improvements in the domains for Body Rocking and Expressionless Face (nominal *p* = 0.0132; Cohen's *d* effect size = 0.39) and Fear/Anxiety (nominal *p* = 0.0003; Cohen's *d* effect size = 0.58). The Cohen's *d* effect sizes reported in the phase 2 study and in LAVENDER were of medium size and comparable to those reported for FDA-approved treatments and suggestive of clinically meaningful changes.

## Conclusions

The RSBQ is a valuable tool for the evaluation of RTT and, as an efficacy outcome measure in clinical trials, is easy to understand and specific to the core features of RTT. The heterogeneity of the domains that constitute the RSBQ total score complements the heterogeneity of findings in RTT and allows the detection of a drug effect whether improvements are observed in one or all of the 8 domains. Given that caregivers are required to complete the assessments in the RSBQ in clinical trials in RTT, and that primary care providers are increasingly expected to engage in routine healthcare assessments for individuals with RTT, it is important that both groups are familiar with the RSBQ and its metrics. Future trials should consider additional assessment tools to augment the domains of the RSBQ such as scales measuring verbal and nonverbal communication and the recently validated Gastrointestinal Health Questionnaire for Rett Syndrome (46).
